# Assessment of serpulid-hydroid association through the Jurassic: A case study from the Polish Basin

**DOI:** 10.1371/journal.pone.0242924

**Published:** 2020-12-09

**Authors:** Jakub Słowiński, Dawid Surmik, Piotr Duda, Michał Zatoń

**Affiliations:** 1 Institute of Earth Sciences, University of Silesia in Katowice, Sosnowiec, Poland; 2 Faculty of Computer Science and Materials Science, University of Silesia in Katowice, Chorzów, Poland; Uniwersytet Warszawski, POLAND

## Abstract

The coexistence of sessile, tube-dwelling polychaetes (serpulids) and hydroids, has been investigated. Serpulid tubes bearing traces after hydroids are derived from different stratigraphic intervals spanning the Middle and Upper Jurassic, the rocks of which represent the diverse paleoenvironments of the Polish Basin. Although fossil colonial hydroids classified under the species *Protulophila gestroi* are a commonly occurring symbiont of these polychaetes during the Late Cretaceous and Cenozoic, they seem to be significantly less frequent during the Jurassic and limited to specific paleoenvironments. The hydroids described here are represented by traces after a thin stolonal network with elongated polyp chambers that open to the outer polychaete tube’s surface with small, more or less subcircular apertures. Small chimney-like bulges around openings are an effect of the incorporation of the organism by *in vivo* embedment (bioclaustration) within the outer layers of the calcareous tube of the serpulid host. Considering the rich collection of well-preserved serpulid tubes (>3000 specimens), the frequency of bioclaustrated hydroids is very low, with an infestation percentage of only 0.6% (20 cases). It has been noticed that only specimens of the genus *Propomatoceros* from the Upper Bajocian, Lower Bathonian, Middle Bathonian, and Callovian have been found infested. However, the majority of bioclaustrated hydroids (17 cases) have been recorded in the Middle Bathonian serpulid species *Propomatoceros lumbricalis* coming from a single sampled site. Representatives of other genera are not affected, which is congruent with previous reports indicating that *Protulophila gestroi* was strongly selective in the choice of its host. A presumably commensal relationship is compared with the recent symbiosis between the hydroids of the genus *Proboscidactyla* and certain genera of sabellid polychaetes.

## Introduction

Organisms colonizing other organisms are usually referred to as epibionts ([1]; see also [2]). Such organisms have been present throughout the entire Phanerozoic, colonizing a variety of available hosts (see [2] for a comprehensive review). In many instances, such epibiont-host organism associations were symbiotic (e.g., [3–5]) with a fossil record extending as far back as the Cambrian (e.g., [5–7]).

An immense majority of epibionts bear calcareous skeletons that can easily be fossilized, leaving behind many soft-bodied colonizers unpreserved. However, some soft-bodied organisms adhering to the substrate they colonized may have been preserved due to bioimmuration or bioclaustration (see [8–18]), the processes that provide a unique glimpse of the morphology, diversity, and abundance of tiny, soft-bodied epibionts, which otherwise would have not been fossilized.

One such group of soft-bodied organisms, which are the focus of the present paper, are colonial hydroids symbiotically inhabiting serpulid polychaetes. The hydroids grew simultaneously within the outer layers of infested polychaete tubes, embedded within their skeleton due to the process known as bioclaustration ([10]; see also [11, 17, 19, 20]). Although the polychaete–hydroid association has already been noticed by J.D.C. Sowerby [21], Rovereto [22] was the first to describe it. Based on the material from the Pliocene of Italy, Rovereto [22] classified the bioclaustrated hydroids as *Protulophila gestroi*. However, despite the fact that the name was adopted for the fossil, his taxonomic interpretation was incorrect, as he inferred the traces of an organism to be a new genus and species of ctenostomatous bryozoan being adherent to the surface of a tube belonging to the serpulid species *Protula firma*. Further discussions of the affinities of *Protulophila gestroi* did not reveal the true origin of an organism, including interpretations such as the formation of molds on the surface of the tube by a serpulid itself [23] and describing it as a new species of bryozoans [24].

The first full description of the nature of the relationship as well as a detailed description of the fossil itself, which allowed to unequivocally classify the organism taxonomically, was provided by Scrutton [8]. He proved that colonial hydroids infested certain species of tube-dwelling polychaetes and concluded that the first available name for the molds left by the bioclaustrated hydroids is *Protulophila gestroi* Rovereto, 1901. Since then, serpulid–hydroid associations have been reported from sedimentary rocks of different ages, beginning from the Lower Jurassic (Pliensbachian, see [25]), Middle to Upper Jurassic [14, 26], and Cretaceous [15, 27–29]. Interestingly, in all these cases, the hydroid bioclaustrations were invariably classified as a single species–*Protulophila gestroi*. It must also be mentioned that the existence of Recent examples of *Protulophila gestroi* was mentioned for the first time by Jäger in 1993 [30], but no scientific description has been published so far. Recently, Taylor and co-workers announced that investigation of *Protulophila* infesting serpulids from modern seas is under way [31].

To date, any data concerning the serpulid–hydroid relationship come from single stratigraphic units and/or localities, which limit our understanding of the nature of this association. To fill this gap, in the present study, we decided to conduct a thorough assessment of this relationship through an inspection of rich material of serpulid tubes derived from different stratigraphic intervals of the Middle to Upper Jurassic (Bajocian-Kimmeridgian) deposits, representing diverse paleoenvironments within a single paleogeographic entity–the Polish Basin. Such an approach may allow a better picture of the persistence, abundance, and symbiotic relationship of such an association both through time and across paleoenvironments. Additionally, we traced the morphology of the hydroid symbionts and their relationship with the serpulid hosts using scanning electron microscopy and, for the first time for these fossils, micro-computed tomography methods. The phylogenetic affinity of the Jurassic hydroid symbionts was also discussed.

## Materials and methods

### Materials and their provenance

**Fig 1:**
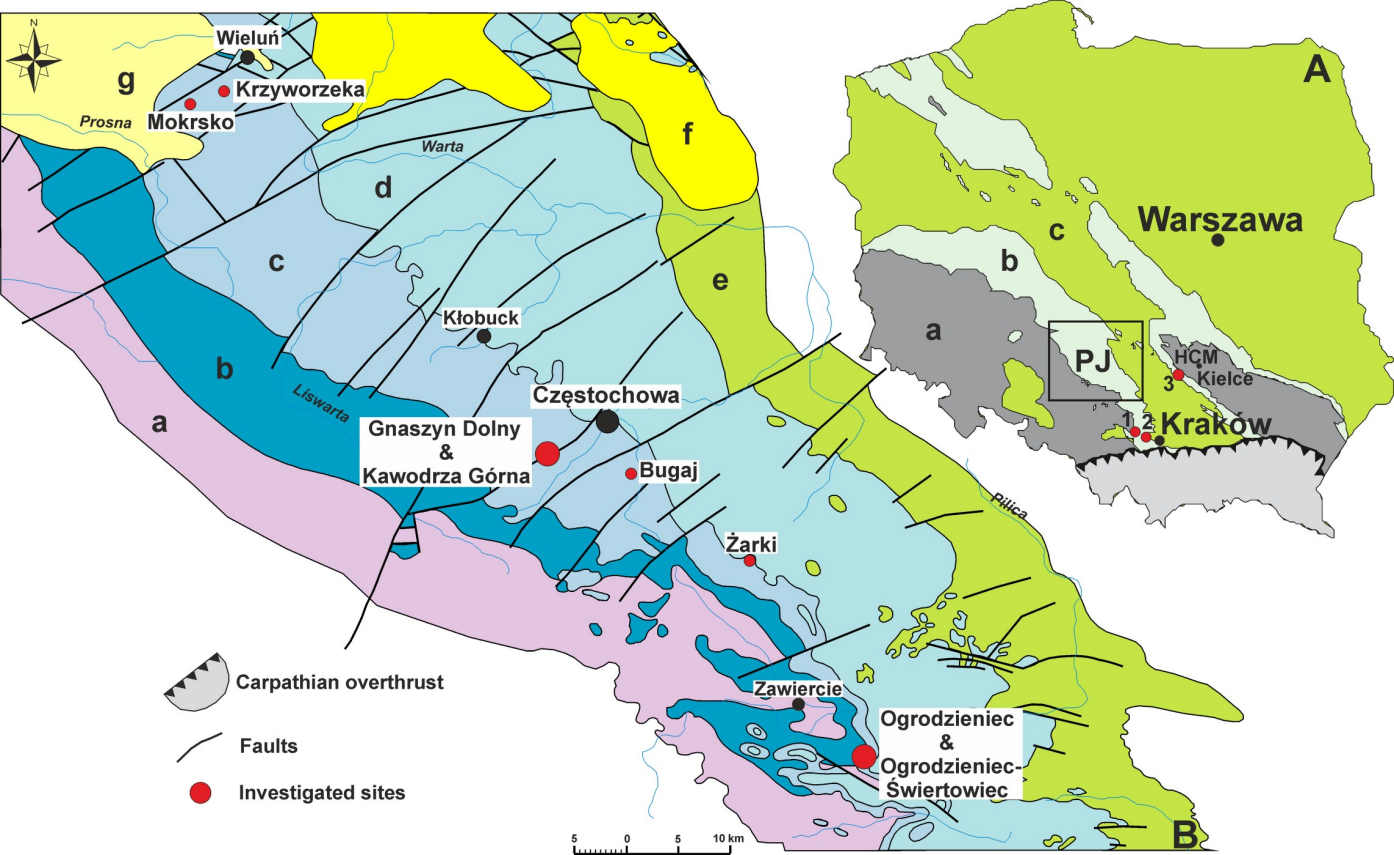
**A**. Geological sketch-map of Poland without the Cenozoic cover with three sampled localities indicated; **a**–pre-Jurassic, **b**–Jurassic, **c**–Cretaceous, HCM–Holy Cross Mountains; PJ–Polish Jura; 1 –Bolęcin; 2 –Zalas; 3 –Małogoszcz. **B.** Geological map of the Polish Jura area without Quaternary cover, with sampled localities indicated; **a**–Upper Triassic, **b**–Lower Jurassic, **c**–Middle Jurassic, **d**–Upper Jurassic, **e**–Cretaceous, **f**–Miocene, **g**–Pliocene (modified after Zatoń et al. [32]).

The fossils discussed herein were derived from eleven outcrops representing different stratigraphic intervals, spanning the Middle to Upper Jurassic, and various marine paleoenvironments of the Polish Basin (Fig 1). All of the fossil material collected is stored at the Institute of Earth Sciences in Sosnowiec (abbreviated GIUS 8–3730).

#### Bolęcin

Bolęcin is located in the area of the Polish Jura about 6 km to the east of the town of Chrzanów (50°06'25"N, 19°29'25"E), between Katowice and Kraków (Fig 1). The fossils collected from this locality have been found in highly fossiliferous, sandy limestones containing abundant non-skeletal grains (such as quartz pebbles and ooids), most probably referring to the so-called “Balin Oolite.” Fossils are irregularly distributed within the deposits. Polychaete tubes are attached to various fossils such as bivalves, gastropods, ammonites, and belemnites, which act as substrates for these episkeletozoans (*sensu* [33]). Using ammonite fauna [34], the Balin Oolite was dated as Upper Bathonian (Retrocostatum and Discus zones) to Lower Callovian (Herveyi, Koenigi, and Calloviense zones), with a possible base of the Middle Callovian (Jason Zone) also present (see [35]). The stratigraphic ranges of diverse ammonite genera and low thickness (less than 1 m) of reworked deposits indicate that the carbonates are condensed, as previously noted by Tarkowski et al. [36] and Mangold et al. [34]. The presence of diverse fauna, including ammonites (e.g., [34]), may indicate an open marine paleoenvironment. In total, 1,011 polychaete tubes were inspected, of which 589 were well-preserved. From now on, the term “well-preserved” refers to those specimens which have unabraded external tube portion showing any signs of bioclaustrated hydroids.

#### Zalas

The material was collected in an active quarry located in the Zalas village near Krzeszowice (50°05'06.7"N 19°38'49.5"E) in the southern part of the Polish Jura (Fig 1). Fossils were derived from both Callovian sandy limestones and overlying Oxfordian deposits. Transgressive Middle Jurassic deposits discordantly overlie uneven Lower Permian porphyres and rhyodacites [37], forming a laccolith [38], which are the major subjects of quarry exploitation. Within the Callovian deposits, sclerozoan hosts occur within a hardground (Middle Callovian and the lower part of Upper Callovian; see [37, 39]), which originated in an open-sea, deep shelf environment [39, 40]. Diverse organisms bearing hard exoskeletons play a role as substrates for diverse and abundant sclerobionts (see [40]). A substantial majority of serpulids have been found fixed to large bivalves *Ctenostreon proboscideum* (J.D.C. Sowerby); however, some of them have also been found encrusting belemnites, ammonites (*Macrocephalites*), and nautiloids. Lower Oxfordian serpulids have been found attached only to sponges that form sponge bioherms (e.g., [41]). In total, 1,684 specimens (976 well-preserved) from both Callovian and Oxfordian were inspected.

#### Małogoszcz

Fossils from Małogoszcz are derived from an active quarry located about 1 km to the north of Małogoszcz town center (50°49'21.6"N 20°15'39.2"E) and about 30 km to the west of the city of Kielce (Fig 1). It is situated in the southwestern part of the Mesozoic Border of the Holy Cross Mountains [42–44]. In this locality, all the collected polychaete tubes encrust bivalve shells, the majority of which belong to the genus *Actinostreon*. The fossil-rich, Lower Kimmeridgian (Hypselocyclum and Divisum zones, see [43–45]) deposits are referred to as the Skorków Lumachelle [43]. The shell-bearing deposits are an effect of storm episodes in a relatively shallow marine environment [43]. In total, 157 serpulid polychaetes (104 well-preserved) encrusting oyster bivalves were examined.

#### Ogrodzieniec

Sediments from Ogrodzieniec, together with the following localities, occur as epicontinental deposits forming monotonous sequences of dark mudstones and siltstones [46–50]. These deposits are referred to as the Ore-bearing Częstochowa Clay Formation (see [46, 51, 52]). These siliciclastics are intercalated with numerous levels of isolated and horizon-forming, carbonate, and fossil-rich concretions (e.g., [51, 53]). This and the following locality are confined to the southern sedimentary region of the Polish Jura, which is considered to represent the shallow, marginal part of the Polish Basin [49, 54]. The deposits originating in the southern sedimentary region are more variable with respect to facies, are thinner, and bear common hiatuses [54], which are also evidenced by widespread exhumed carbonate concretions (hiatus concretions) marking distinct breaks in sediment supply and/or sea-floor erosion (see [26]). In Ogrodzieniec, the fossils were cemented to the hiatus concretions collected in a small clay-pit (50°27'31.1"N 19°30'13.1"E) with an exposure of siltstones of ca. 8 m thick. The concretion-bearing deposits represent the Upper Bathonian, ranging up to its topmost Discus Zone, as evidenced from dinocysts [55]. In total, 337 serpulid fossils (122 well-preserved) were inspected.

#### Ogrodzieniec-Świertowiec

The specimens collected were derived from a small outcrop located approximately 1 km south of the town center of Ogrodzieniec (50°26'23.8"N 19°31'13.0"E). Condensed, sandy, and carbonate sediments underlying the dark mudstones of the ore-bearing Częstochowa Clay Formation are exposed (e.g., [48, 56]). Polychaete tubes form dense aggregations on the surface of large oncoids. Due to the occurrence of dinoflagellate cysts (*Valansiella ovula* and *Ctenidodinium* cf. *combazii*, Przemysław Gedl, written communication, March 2019), foraminifers *Paleomilliolina czestochowiensis* (Zofia Dubicka, written communication, March 2019) found within the oncoid cortices [57], and previously noted ammonites *Parkinsonia* spp. and *Parkinsonia* (*Oraniceras*) *gyrumbilica* (see [48]), the condensed oncoid-bearing interval is confined to the Upper Bajocian–Lower Bathonian (up to the Macrescens Subzone of the Zigzag Zone). Considering the cyanobacterial genesis of the oncoids [56, 57], serpulid polychaete worms inhabited photic conditions, slightly beneath the fair weather wave base. In total, 1,103 specimens (621 well-preserved) were examined.

#### Żarki

Deposits in Żarki, together with all the following localities, are confined to the northern sedimentary region of the Polish Jura, which is characterized by thicker and more complete sequences of ore-bearing clays [54]. Sediments within these localities are interpreted to have been deposited in calm, epicontinental paleoenvironments with generally well-oxygenated bottom waters (e.g., [49, 58–60]). However, some ichnofabrics, accumulations of shell detritus, and erosional surfaces indicate episodic storm events [61]. All of the fossils investigated herein are attached to carbonate hiatus concretions. In this locality (an active brick-pit, 50°37'09.0"N 19°22'02.7"E), concretions were partly collected *in situ* from the bottom part of a 15 m thick section [62]. The age of the concretion-bearing horizon is Upper Bathonian (Hodsoni Zone, see [47]). In total, 58 polychaete tubes have been investigated, of which 23 are well-preserved.

#### Bugaj

In this locality, serpulid polychaetes inhabited Middle Bathonian (likely Morrisi Zone) carbonate hiatus concretions (see [26]). Currently, the excavation in Bugaj (50°45'51.5"N 19°10'05.9"E) is abandoned, and the clay-pit filled by dumped waste material is unavailable for field research. Therefore, the collection of fossils from Bugaj is scientifically valuable. In total, 553 fossils (170 well-preserved) were collected and inspected from this locality.

#### Gnaszyn Dolny

The fossils are derived from the Middle Bathonian (Morrisi Zone) mudstones exposed in the lower part of the “Gnaszyn” brick-pit [26] (50°48'12.6"N 19°02'26.8"E). The serpulid tubes are preserved on bivalve shells (mainly oysters), where they form densely packed aggregations. Clay sediments containing encrusted shells were deposited in a deeper marine paleoenvironment, below the storm wave base (e.g., [26, 63, 64]. In this locality, 777 polychaete tubes have been found, of which 374 are well-preserved.

#### Kawodrza Górna

The specimens collected in Kawodrza Górna (“Sowa” brick-pit, 50°47'05.1"N 19°02'35.4"E) are from the Lower Bathonian deposits (Zigzag Zone, e.g., [46, 53]), which represent similar siliciclastic facies and paleoenvironment as those present in neighboring Gnaszyn Dolny (e.g., [46, 64]). All of the fossils found encrust oyster shells. In total, 90 polychaete tubes were collected (57 well-preserved).

#### Mokrsko

Polychaete tubes from this locality commonly encrust carbonate hiatus concretions occurring in the “Mokrsko” brick-pit (see [26], 51°10'00.4"N 18°26'05.7"E). The concretions form a continuous horizon; however, some of them are irregularly distributed within glaciotectonically deformed clay sediments [26]. Due to the presence of the ammonites *Parkinsonia* [32], the age of sediments is Upper Bajocian (Parkinsoni Zone). The deposits in Mokrsko, as in the case of the above-mentioned deposits, represent a paleoenvironment probably located below the storm wave base [26]. In total, 358 specimens (111 well-preserved) were inspected.

#### Krzyworzeka

At this locality (51°10'06.9"N 18°31'07.1"E), all the collected polychaete fossils encrust hiatus concretions; however, the majority of them are strongly abraded. Krzyworzeka is the northernmost locality in the area investigated and the sediments thought to have been deposited in a calm environment related to an outer shelf (e.g., [26, 65]), located below the storm wave base. However, due to the overturning of the concretions, episodic storms are not excluded (see [26]). Dinoflagellate cyst dating indicates that deposits from Krzyworzeka range up to the Upper Bathonian Discus Zone [66]. In total, 920 specimens (199 well-preserved) were inspected.

## Methods

All the specimens derived from the localities listed above were carefully inspected under a binocular microscope, paying special attention to the potential occurrence of bioclaustrations after hydroids. Although all serpulid tubes in each locality were counted, we separately counted unabraded, externally well-preserved tubes, and poorly-preserved specimens, having abraded or strongly exfoliated tubes showing no signs of bioclaustration. Those tubes bearing hydroid symbionts were determined to at least the generic level.

Polychaete tubes bearing hydroid traces were cleaned using an ultrasonic cleaner, and selected specimens were examined under an environmental scanning electron microscope (ESEM) Philips XL30 at the Institute of Earth Sciences in Sosnowiec. The fossils were scanned in an uncoated state in back-scattered electron (BSE) imaging mode.

Four serpulid tubes (two from Gnaszyn Dolny, one from Kawodrza Górna, and one from Mokrsko) with the best-preserved and visible bioclaustration traces were selected for further examination using computed microtomography (micro-CT). Virtual sections were made in the X-ray Microtomography Laboratory at the Faculty of Computer Science and Materials Science, University of Silesia in Katowice, Chorzów, Poland, using the GE Phoenix v|tome|x micro-CT equipment with scanning voltage ranging 140–180 kV, current intensity 50–90 μA, and scanning time of 25 to 35 min depending on the sample. The collected images were processed using Volume Graphics® VGSTUDIO Max software and Volume Graphics® myVGL Viewer App and Fiji [67]. Based on the micro-CT scans, a volumetric rendering and movie were produced using Drishti [68].

## Results

### Morphology of the bioclaustrated hydroids

The bioclaustrated traces after hydroids referred to as *Protulophila gestroi* are represented by systems of stolons and polyp chambers (reflecting casts of zooids) preserved by the skeletal overgrowth of the host serpulid polychaetes. Thus, the structures preserved within the serpulids investigated here are in agreement with other hydroid bioclaustrations reported so far.

**Fig 2:**
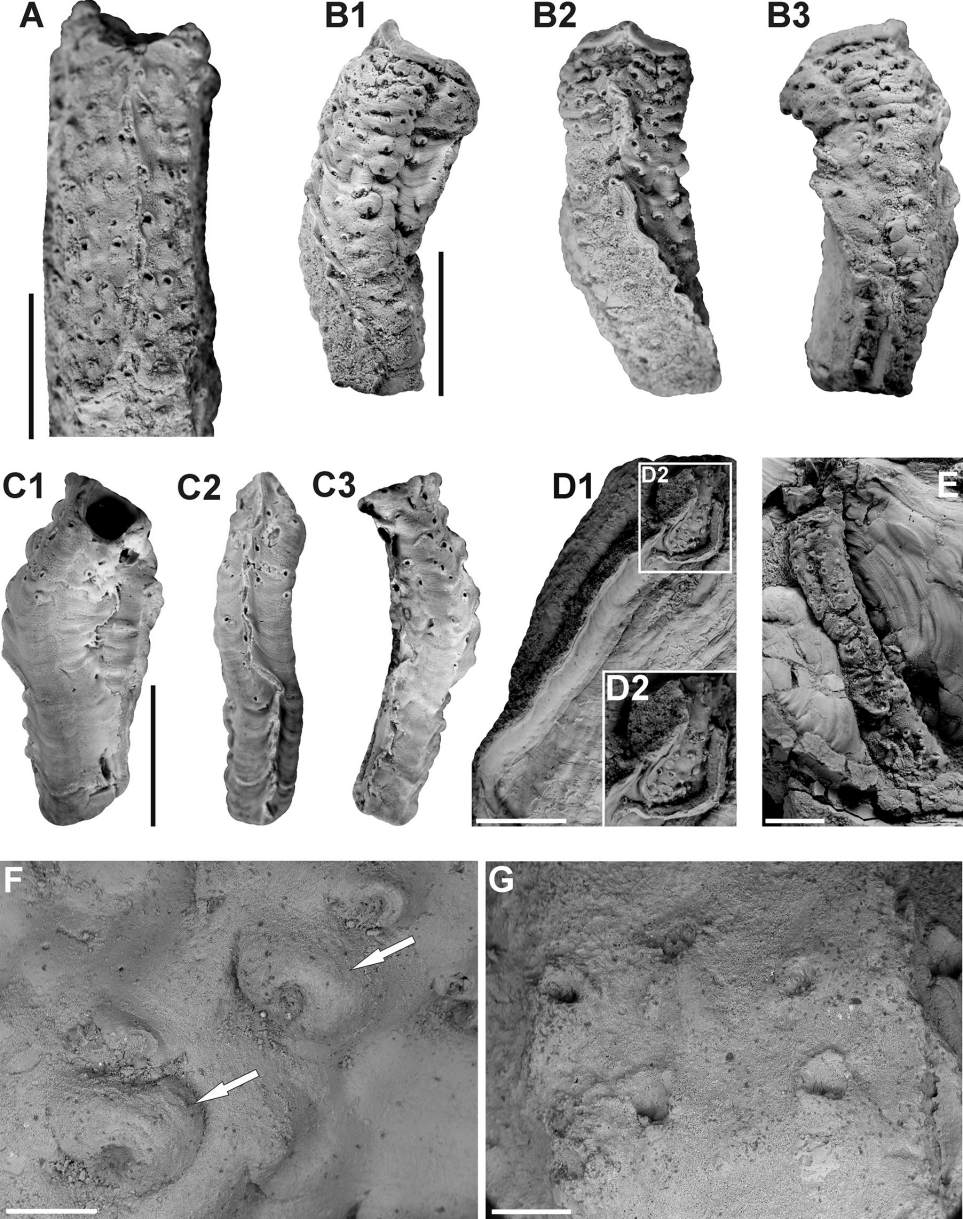
*Protulophila gestroi* Rovereto bioclaustrated by two species of the serpulid *Propomatoceros* from the Polish Jura. **A.** A strongly infested *Propomatoceros* sp. from the Upper Bajocian of Mokrsko, exhibiting a regular arrangement of the colony. External appearance shows apertures slightly bent toward the anterior of the tube; elongation of polyp chambers to the serpulid’s growth direction is visible, GIUS 8-3730/1. **B.** Strongly infested *Propomatoceros lumbricalis* (von Schlotheim) from the Middle Bathonian of Gnaszyn Dolny. Dense arrangement of the hydroid colony shows different apertural morphologies within one specimen. Three views (two lateral and one dorsal) show hydroid colony embedment around nearly entire tube, GIUS 8-3730/2. **C.** Infested *Propomatoceros lumbricalis* from the Lower Bathonian of Kawodrza Górna, showing an irregular colony pattern with small polyp openings scattered over the tube. Aperture lips are slightly flattened, forming only indistinct hoods. A hydroid colony encircled the whole tube from the base to the keel (as shown in three views of the tube), GIUS 8-3730/3. **D.** A hydroid colony located in the anterior part of the tube of *Propomatoceros lumbricalis* from the Middle Bathonian of Gnaszyn Dolny. Single apertures are bent backward, GIUS 8-3730/4. **E.** Moderately infested tube from the Middle Bathonian of Gnaszyn Dolny with a relatively regular colony pattern, GIUS 8-3730/5. **F-G.** ESEM back-scattered images presenting detailed morphology of hydroid apertures. In **F**, two robust bosses overhanging the apertures (indicated by white arrows) and a few smaller hoods are shown, GIUS 8-3730/2. **G** indicates delicate lumps, GIUS 8-3730/1. Scale bars: 5 mm **(A-E)**, 0.5 mm **(F-G)**.

**Fig 3:**
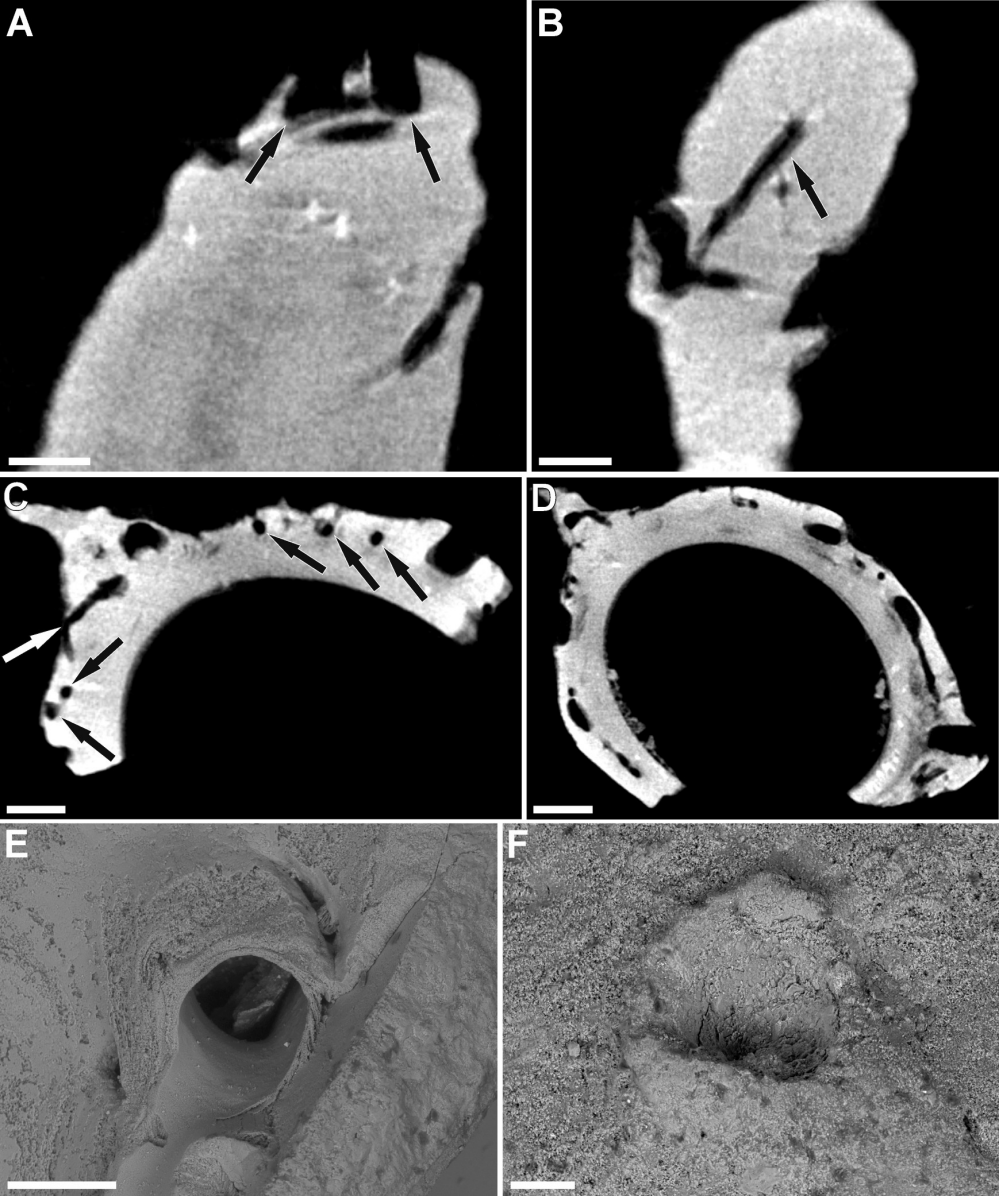
Internal appearance of serpulid-hydroid association shown on micro-CT scans **(A-D)** and ESEM back-scattered images **(E-F)**. **A.** Scan of the serpulid tube from the Lower Bathonian of Kawodrza Górna (the lateral view of the tube); black arrows show two polyp chambers with the depth of embedment visible, GIUS 8-3730/3. **B.** Scan of the tube from the Middle Bathonian of Gnaszyn Dolny; black arrow indicates the longitudinal section of the stolonal tube, GIUS 8-3730/2. **C-D.** Cross-section scans of the infested serpulid tube from the Lower Bathonian of Kawodrza Górna, GIUS 8-3730/3. In **C,** the arrows show the stolonal tubes in cross-section (black arrows), and longitudinal (slightly inclined) section (white arrow). In **D,** cross-sections of polyp chambers are visible, as well as the branching stolonal tubes connected to them. **E-F.** Single hydroid apertures. **E.** Specimen from the Lower Bathonian of Kawodrza Górna, GIUS 8-3730/3. **F.** Specimen from the Upper Bajocian of Mokrsko, GIUS 8-3730/1. Scale bars: 0.25 mm **(A-C)**, 0.35 mm **(D)**, 0.2 mm **(E)**, 0.1 mm **(F)**.

The external appearance of the hydroids dwelling within the serpulid’s tube is shown by the presence of small, more or less subcircular apertures superficially resembling borings (see [10]), and chimney-like bosses scattered both regularly and irregularly over the serpulid tube’s exterior (Fig 2A–2E). With a few exceptions, the apertures are declivous toward the anterior of the tube with a proximal lip slightly flattened, and distal lip curved and uplifted, often forming a small hood, or a bigger, irregular lump (Fig 2). In some cases, the apertures are gently bent in different directions, which might reflect the adjustment of zooids to the ontogenic skeletal growth of the tube as well as to its bulges and curvatures (Fig 2B and 2D). Due to the increasing stolonal network depth of burial and the rugosity of a tube, bosses become bigger, more robust and solid, and occasionally overhang orifices (Fig 2B and 2F). A large majority of these small polyp openings, exhibiting the external appearance of the particular zooids, is located in the anterior part of the dwelling tube (Fig 2A–2D). None of the specimens of *Protulophila gestroi* have been found bioclaustrated in the tube’s posterior (however, one specimen is represented by a fragmented tube with indistinct characters providing recognition of the part of the tube). Some hydroid colonies encircle nearly a whole serpulid tube, occupying the surface from the keel on the top to the same base (Fig 2B and 2C). Both the shape and size of the apertures are variable, even within the same specimen. The orifice size ranges from 0.15 to 0.25 mm in diameter (Fig 3E and 3F), with bosses up to even 1 mm across.

**Fig 4:**
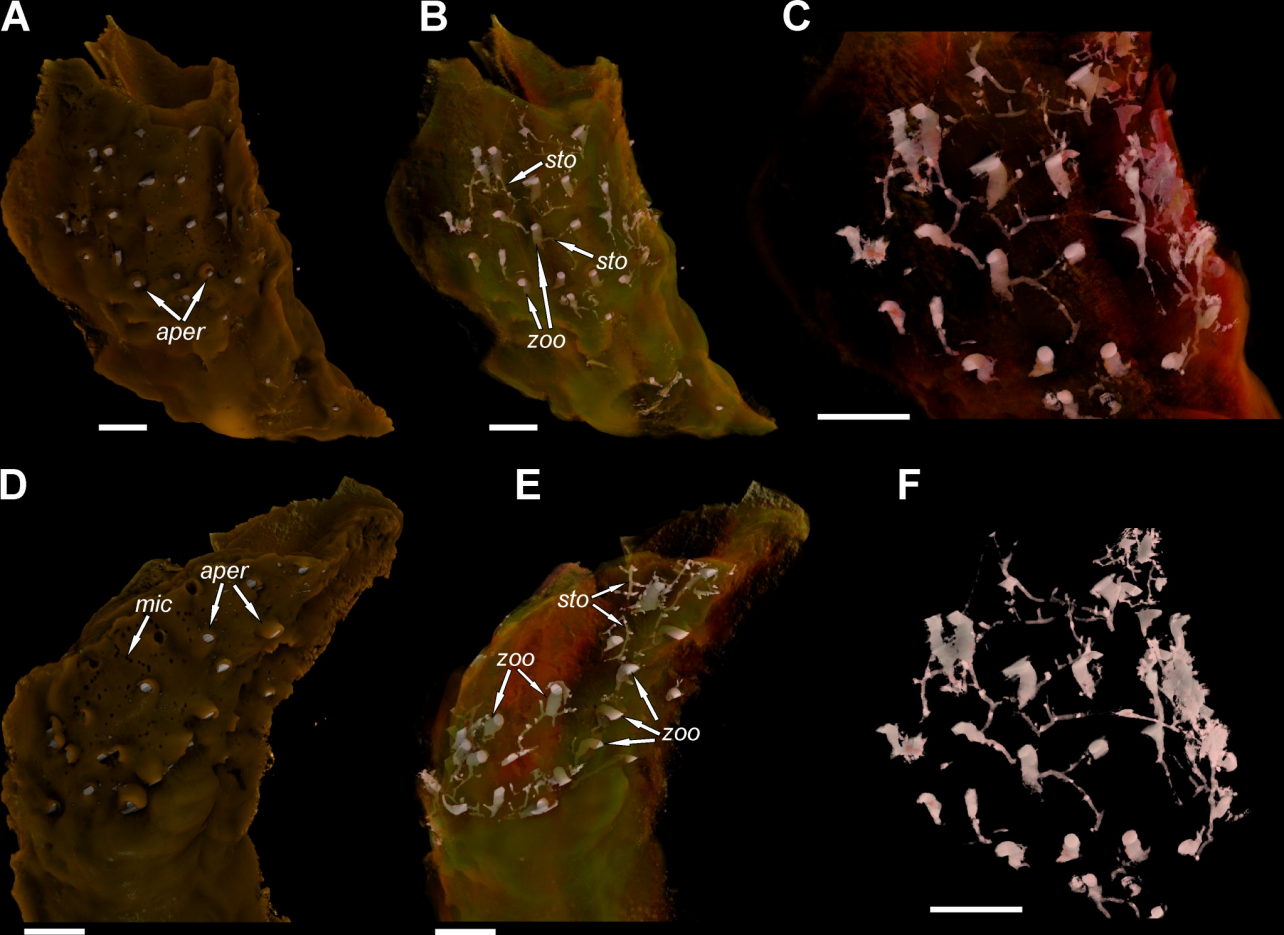
Visualization of the bioclaustrated hydroid colony *Protulophila gestroi* by the host serpulid *Propomatoceros lumbricalis* from the Middle Bathonian of Gnaszyn Dolny, GIUS 8-3730/8. **A**. External left side of the tube showing bioclaustrated apertures after hydroids (*aper*). **B-C**. The same side showing the morphology and internal arrangement of hydroid zooids (*zoo*) and stolons (*sto*), magnified in **C**. **D**. Right side of the tube showing bioclaustrated apertures after hydroids (*aper*) and numerous microborings (*mic*). **E**. The same side showing the morphology and internal arrangement of hydroid zooids (*zoo*) and stolons (*sto*). **F**. Isolated system of stolons and hydroid zooids embedded within the tube showed in **C**. Serpulid aperture is on the top. Scale bars: 1 mm (**A-F**).

Based on the micro-CT scans and resulting visualization (Fig 4), an internal appearance of this association shows a network of branching stolonal tubes embedded in the tube wall, joining together in the hydroid’s chambers (Fig 4B, 4C, 4E and 4F). These elongated, cylinder-shaped internal cavities after particular polyps, well-visible in the visualization (Fig 4), are buried concurrently to the surface of a tube and are bent outward, which is revealed on the external part of the tubes by small orifices (Fig 4). Polyp chambers are elongated in the direction of the skeletal growth of the worm (Fig 2A, 2E and 2G). The mean chamber size is 0.84 mm long and 0.31 mm wide, while stolons have a diameter of 50 μm (Fig 3A–3D). Depending on the depth of embedment, the stolonal network is visible in some specimens, being embedded relatively shallowly in the external part of a tube (Figs 2C and 3A). The irregular arrangement of the colony in some cases might be a result of the tube’s external morphology or inconstant skeletal growth. However, despite the differences in particular hydroid’s stolonal network depth within the serpulid tube, it tends to be incorporated at an approximately constant depth in the tube’s interior within the same specimen of the host serpulid. The depth of hydroid network embedment varies from 0.19 to 0.63 mm, depending on the specimen (Fig 3A).

### Frequency and occurrence of bioclaustrated hydroids

Following an examination of 7,048 specimens of tube-dwelling polychaetes, of which 3,346 are well-preserved, biocluastration traces after the hydroids *Protulophila gestroi* have been noted in only 20 specimens of serpulid tubes (Table 1). Thus, in the present case, the overall percentage of hydroid infestation was notably low, with a rate of only 0.28% among all the specimens of polychaete tubes collected. Although the percentage of infestation among the well-preserved fossils is more than twice as high, at 0.6%, this value is very low. However, due to the significant abrasion of the tubes acting as a substrate for *Protulophila gestroi*, potentially embedded hydroids, if they ever existed, would not have been preserved in the fossil record. Thus, it is possible that the real percentage of hydroid infestation might have been higher than presented herein.

**Table 1:** Data on stratigraphy, provenance, encrusted substrate type, number of all and well-preserved serpulid tubes, and number of tubes infested by hydroids.

	Stratigraphic interval												
	Upper Bajocian	Lower Bathonian	Middle Bathonian	Middle Bathonian	Upper Bathonian	Upper Bathonian	Upper Bathonian	Bajocian-Bathonian	Upper Bathonian-Lower Callovian	Callovian	Oxfordian	Lower Kimeridgian	
	**Locality**												
**Kind of substrate**	Mokrsko	Kawodrza Górna	Gnaszyn Dolny	Bugaj	Ogrodzieniec	Krzyworzeka	Żarki	Ogrodzieniec-Świertowiec	Bolęcin	Zalas	Zalas	Małogoszcz	
Bivalves		57	220						283	667		104	
		33	262						255	413		53	
Belemnites			154						22	15			
			141						9	11			
Hiatus concretions	111			170	122	199	23						
	247			383	215	721	35						
Oncoids								621					
								482					
Sponges											249		
											261		
Ammonites									33	8			
									16	12			
Nautiloids										37			
										11			
Gastropods									251				
									142				
**No. specimens**	358	90	777	553	337	920	58	1,103	1,011	1,174	510	157	**7,048**
**Well-preserved specimens**	111	57	374	170	122	199	23	621	589	727	249	104	**3,346**
**No. hydroids**	**1**	**1**	**17**	0	0	0	0	0	0	**1**	0	0	**20**

Serpulid tubes played the role of a substrate for colonial hydroids, potentially providing both insight into the paleoecological issues and information about the relationship. Of all the polychaete tube fossils examined, only specimens assigned to the genus *Propomatoceros* Ware have been found to be infested by *Protulophila gestroi*, the data on which is presented in Table 2. The tubes of *Propomatoceros* investigated here possibly represent two species: 1) slender, gracile forms with distinctive longitudinal keels on the top of the Lower Bathonian of Kawodrza Górna and Middle Bathonian of Gnaszyn Dolny, referred to as the species *Propomatoceros lumbricalis* (von Schlotheim) on the basis of similarity to the Middle Jurassic species described by Ippolitov [69], and 2) larger, robust tubes from the Upper Bajocian of Mokrsko and Callovian of Zalas, referred to as the species *Propomatoceros* sp. The tubes may be slightly curved, increasing in diameter toward the anterior part (faster in *Propomatoceros lumbricalis*), with a subtriangular cross-section (more distinct in *Propomatoceros lumbricalis*). The external surface is relatively smooth and sometimes uneven with very small bulges. Attachment structures are usually well-developed and visible. The arrangement of the hydroid colonies seems to be more regular within the species *Propomatoceros* sp.

**Table 2:** Data on the occurrence of bioclaustrated hydroids on the background of the investigated *Propomatoceros* tubes.

	Mokrsko	Kawodrza Górna	Gnaszyn Dolny	Zalas
	(Upper Bajocian)	(Lower Bathonian)	(Middle Bathonian)	(Callovian)
**Well-preserved specimens**	9	16	187	52
**Poorly preserved specimens**	15	5	74	36
**No. hydroids**	**1**	**1**	**17**	**1**
**% infestation of well-preserved specimens**	11,11%	6,25%	9,09%	1,92%

The other serpulid taxa present on the Upper Bajocian through Kimmeridgian substrates investigated here (see [26, 56, 70, 71]), such as *Nogrobs*, *Cementula*, *Filogranula*, *Spiraserpula*, *Metavermillia*, *Mucroserpula*, *Placostegus*, and the most abundant species *Glomerula gordialis*, lack any traces after bioclaustrated hydroids. Moreover, 17 out of 20 specimens of *Protulophila gestroi* have been found only on the tubes coming from a single locality and stratigraphic interval (Gnaszyn Dolny, Middle Bathonian, Morrisi Zone, Table 2).

## Discussion

### Morphology and phylogenetic affinity of *Protulophila gestroi*

The overall pattern of the colonial hydroids clearly indicates that stolonal network and polyp chambers were incorporated *in vivo* within the external parts of the host’s tube during its skeletal growth and, as previously recognized by Scrutton [8], cannot be considered as a boring activity. Deflected apertural lips (marked by the differential rate of skeletal secretion of the host) and the fact that none of the polyp orifices is fully overgrown by the serpulid (which might have happened after the death of the hydroid) indicate that both the colonizer and the host must have been alive during interaction. The general arrangement of stoloniferous hydroids is more or less convergent with all the reported cases from different stratigraphic intervals (e.g., [8, 14, 15, 18, 25–29]). Slight differences in morphological details may only imply different physiological and/or paleoecological conditions as well as supposedly interspecific variations (see below).

Although the mode of origin of the fossils has already been well recognized by Scrutton [8], with the corroboration of all subsequent reports, the close phylogenetic relationships of *Protulophila gestroi* are still uncertain. In attempts to link *Protulophila gestroi* with contemporary species of hydroids, it is generally affiliated with the living genus *Proboscidactyla* Brandt [72]; however, apart from Scrutton [8], who provided a detailed description of its possible affinity, little attention has been paid to this problem. *Proboscidactyla* is an obligatory inhabitant of sabellid polychaetes (e.g., [73, 74]). This modern hydroid captures nutrients from polychaete-propelled feeding currents and directly from a worm’s radiole [73, 75]. Although this symbiotic relationship is well recognized and has been described several times (e.g., [73, 74, 76]), more attention has been paid to the life cycle and ecology of the medusa form of *Proboscidactyla* (e.g., [77–80]) than to the symbiotic association itself. The comparison of Scrutton [8] seems to be appropriate in terms of lifestyle; however, the overall arrangement of the colony differs strikingly from the colonial pattern of *Protulophila gestroi*. It might be putatively explained as evolutionary changes, from a simple, relatively regular pattern of *Protulophila* to more complex, anastomosing stolons and polyps of *Proboscidactyla*. Moreover, due to taphonomic processes, the morphology of the stolonal network and tentacles of the living organism cannot be directly compared to the fossil preserved solely as an embedded cast. Scrutton [8] also mentioned *Tubularia* as an example of hydroids with a colony growth pattern similar to that observed in *Protulophila gestroi*. However, except this feature, the ecology of *Tubularia* [81] in no way resembles that of *Protulophila gestroi*.

**Fig 5:**
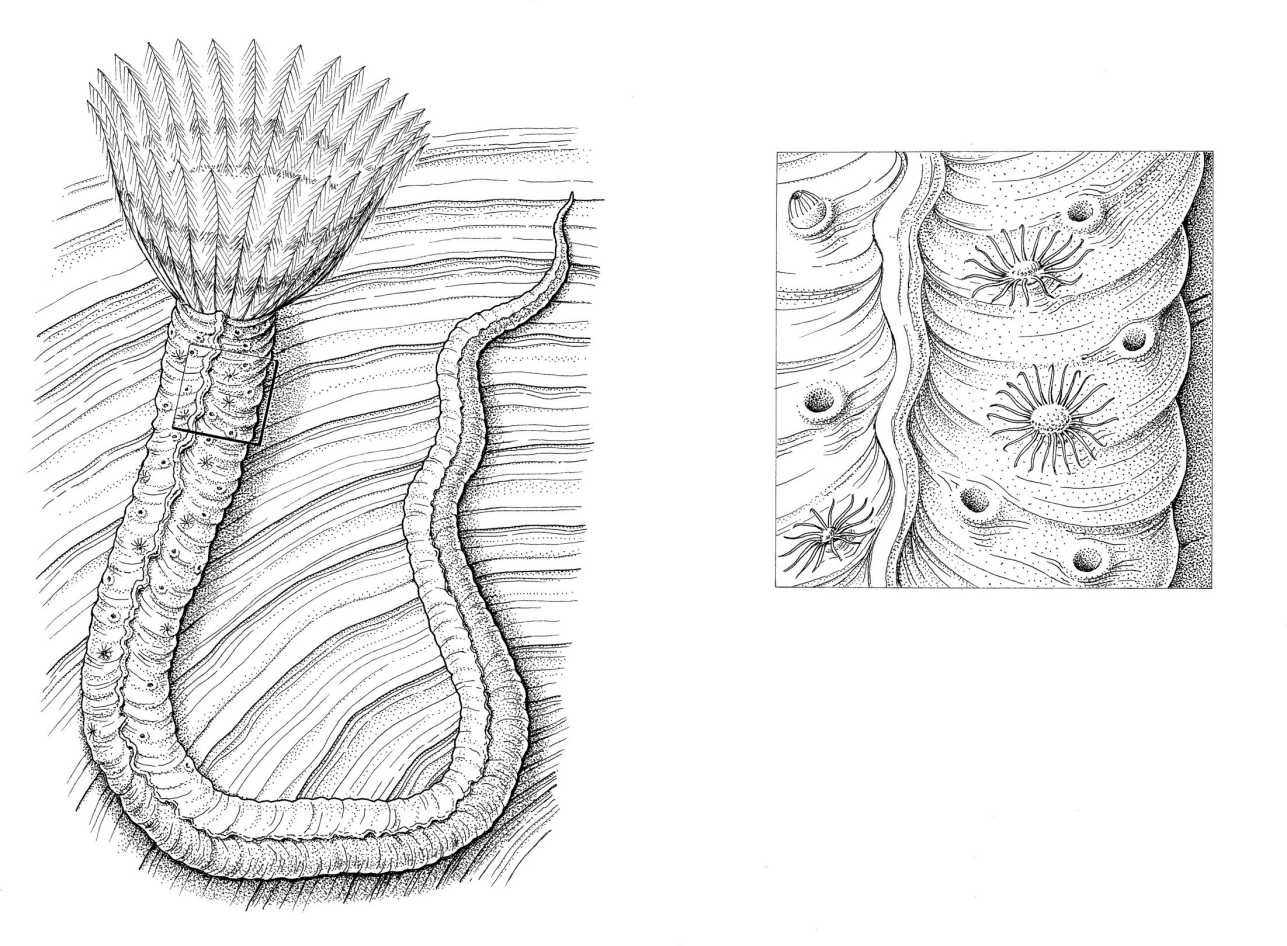
Artistic reconstruction of a Middle Jurassic serpulid *Propomatoceros lumbricalis syn vivo* infested by hydroids *Protulophila gestroi*. It is hypothesized that the everted polyps in older part of the serpulid tube may have relied on the food particles delivered by external currents. An inset shows some everted polyps from the host serpulid’s tube (drawn by Bogusław Waksmundzki).

Reports on the arrangement of *Protulophila gestroi* colonies, with the majority of them located in the anterior parts of polychaetes’ tubes (e.g., [8, 15, 27]; the present study) is convergent with the manner of *Proboscidactyla* colony [73, 75], suggesting a similar lifestyle. Only Zágoršek et al. [29] reported Cretaceous *Protulophila gestroi* as being located in the middle part of the serpulid’s tube, which was interpreted as a growth ceasement before the serpulid’s death [29]. Located close to the tube’s rim, hydroids could have captured food particles from the feeding currents generated by the worm or directly from the brachial crown. The direction of hydranths’ growth (hence the resulting hoods and apertural shape as evidenced in the fossils described here, see Fig 2A–2C and 2F) toward the proximal parts of the tube may also express an attempt to be located closer to the “feathery” radiole of the polychaete and its propelled feeding currents bearing nutrients (Fig 5). However, single apertures have also been found to be bent backward–some hydranths’ growth direction might have been influenced by external currents as well. The second important advantage gained by these hydroids was the protection provided by a hard, mineralized tube of serpulid, which probably became the only profit during astogeny, as the older hydranths gradually receded from the polychaete’s radiole and thus were excluded from the benefits of the host’s feeding currents. Such older hydranths could have been active, anyway, gathering the food delivering by external currents (Fig 5), which is evidenced in the apertures bent backward, a feature which was mentioned above.

Although we cannot phylogenetically link exclusively modern *Proboscidactyla* with the fossil hydroid with full confidence, all of the data collected hitherto on both symbionts seem to be sufficient to provide a reliable comparison, at least in the means of their lifestyles. The possible affinity is strengthened by the bilateral symmetry of apertures of Jurassic hydroids, as suggested by Baliński et al. [82]. However, the phylogenetic affinity of modern *Protulophila* is still under study [31] and, therefore, nothing more can be added here at the moment.

Morphological disparities between specimens of both the external and internal appearance of colonial hydroids are also presumably a reflection of different ecological issues, such as the host’s calcification rate, which directly reflects the external appearance of a hydroid colony. Individual zooids might have adjusted to the skeletal growth of the tube, varying on the calcification activity of the serpulid (see Scrutton’s experiment [8]). Polyps most likely changed their growth orientation to avoid a complete embedment due to calcium carbonate tube precipitation (for the serpulid skeletal formation, see e.g., [83, 84]). Changes in skeletal activity may also be reflected in the depth of burial of the stolonal network (see [8]) and density of the colony. Some morphological modifications potentially may also correspond to the functional specialization of single zooids. According to modern *Proboscidactyla* [73], ancient hydroids might have also possessed different kinds of individuals playing various roles in a colony, such as gastrozooids, gonozooids, and dactylozooids.

**Fig 6:**
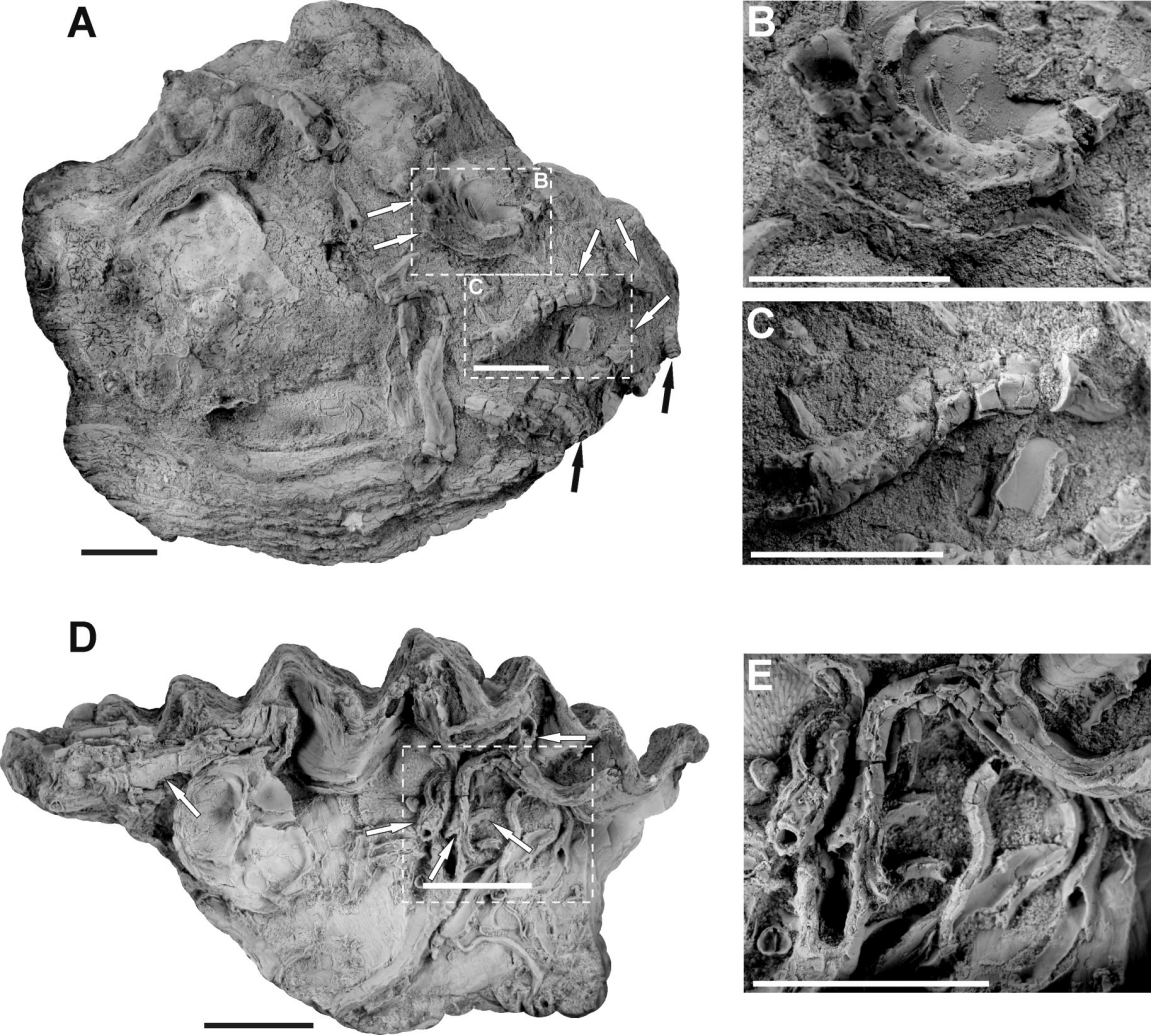
Aggregation of several serpulid tubes on two single oyster valves from the Middle Bathonian of Gnaszyn Dolny, Polish Jura, a number of which (arrowed) possess traces after bioclaustrated hydroids. **A.** An aggregation of six (arrowed) serpulids with *Protulophila gestroi*, GIUS 8-3730/6. **B-C.** Magnified shell areas showing bioclaustrated serpulid tubes, GIUS 8-3730/6a and GIUS 8-3730/6b, respectively. **D.** Aggregation of five (arrowed) serpulid specimens with *Protulophila gestroi*, GIUS 8-3730/7. **E.** Magnified part showing very dense aggregation of tubes on a small substrate surface bearing indistinct bioclaustrated traces (GIUS 8-3730/7a). Scale bars equal 1 cm.

The overall appearance of the fossils studied is relatively similar to all previous reports. Disparities are slight: from elongated orifices with broad sinuses and flattened lips (rare in the present study, see [8], Pl. 39, Figs 1–8; [27, 29]), through some intermediate forms exhibiting delicate hoods (Fig 2A, 2C, 2E and 2G; see also e.g., [8], Pl. 39, Fig 11, Pl. 41, Fig 9), to circular, with robust, chimney-like bosses occasionally overhanging apertures (Fig 2B and 2F; see also e.g., [8], Pl. 39, Figs 13–14; [15], Fig 4A–4D). The size of the polyp opening and density of the colony is variable, as well as the total number of apertures and different tube area infested–either encircling the tube from the keel to its flanges or being scattered more irregularly. The presence of morphological disparities within all the stratigraphic intervals suggests that morphology itself is not particularly useful in taxonomic classification, as it presumably represents differences in the calcifying activity of polychaetes and other ecological factors. As not all *Protulophila gestroi* bioclaustrations reported so far are as well-preserved as those present in some exfoliated serpulid tubes illustrated by Zágoršek et al. [29], the best solution for proper comparison of different colonies, is a micro-CT scanning of various specimens. Further visualization, as made for the present paper, may be helpful in better recognition of key features which then could be used for taxonomic differentiation of particular colonies.

### Selection of the host by hydroids

*Proboscidactyla* is distinctly selective in the choice of the host, often restricted to one or very few species of polychaetes [73, 75]. This may at least partially explain why hydroids (all assigned to *Protulophila gestroi*) spanning from the Pliensbachian [25] to the Pliocene [8, 85], with possible modern representatives [31], have often been described to be selective in their choice of the host, predominantly infesting very few, or even one serpulid species within a given stratigraphic unit and geographic area. Scrutton [8] conducted a comprehensive review of the previously reported Jurassic, Cretaceous, and Cenozoic serpulids from Europe and the Middle East acting as hosts for symbiotic hydroids. All the Jurassic (Bajocian-Oxfordian) hydroids infested different species of his genus *Serpula*, some of which may belong to the genus *Propomatoceros*. Radwańska [14] found only one tube of *Ditrupula* from the Oxfordian of central Poland bearing the hydroids *Protulophila gestroi*. Cretaceous hydroids infested *Rotularia*, *Parsimonia*, *Glomerula* (the only ancient sabellid worm found to be infested), while Cenozoic hydroids were restricted to *Sclerostyla* and *Protula*. Jäger [86] found over 50% of Cretaceous (Coniacian to Upper Maastrichtian) *Martina turbinella* (*Laqueoserpula*?) tubes from northern Germany infested by *Protulophila gestroi*, while other species were clearly more rarely infested. In Poland, Radwańska [27] described hydroids associated with three polychaete species from the Campanian (*Proliserpula ampullacea*, *Pentaditrupa subtorquata*, *Sclerostyla macropus*) and only one from Maastrichtian (*Ditrupula quadrisulcata*). Niebuhr and Wilmsen [15] reported hydroid-bearing serpulids exclusively represented by the genus *Rotulispira* from the Middle Cenomanian of northern Germany. Kamali Sarvestani et al. [18] also reported infested *Rotulispira* serpulids from the Lower-Middle Cenomanian of Iran. All the current data from the Middle and Upper Jurassic of Poland presented here clearly show that each encountered *Protulophila gestroi* colony is associated with a single genus *Propomatoceros*, of which most often infested (18 cases) is the species *Propomatoceros lumbricalis*. Interestingly, Ippolitov [69] also mentioned that the latter serpulid species was most often colonized by *Protulophila gestroi* in the Middle Jurassic (Callovian) of Russia.

It seems less likely that a single (as stated here) species of hydroid, generally described as a selective in its choice of the host, had colonized many diverse species of polychaete worms through such a long geologic time (ca. 190 Ma) in different locations around the world. Comparing this with *Proboscidactyla*, where a variety of species are found fixed to different sabellids [73], it seems to be even more hesitant. The general pattern of *Protulophila gestroi* shows only slight morphological disparities (as described above) among all, very extensive fossil records, and none specific characteristics of fossils seem to correspond with any specific stratigraphic interval. Here appears a dilemma: despite the high probability of the existence of more than one species within all specimens assigned to *Protulophila gestroi*, we are not able to distinguish potentially separate species for taphonomic reasons and the resulting scarce, insufficient data of indistinct modifications of these fossils, regardless of the geological time. Thus, the putative interspecific variation of *Protulophila gestroi* is very difficult, or even impossible, to assess objectively based on the external morphology alone.

The settlement preferences of hydroids throughout the Mesozoic and Cenozoic surely indicate some favorable conditions for the settler. A possible factor strongly influencing larval recruitment and subsequent bioclaustration is a conducive chemical composition. The settling of a juvenile form and its subsequent development may have also been enhanced by physiological and behavioral factors (see [87, 88]), some of which may be unobtainable from the fossil record. Allegedly, the selectivity of hydroid could even concern particular specimens, for example, due to a particular characteristic of the potential host providing a stable substrate to anchor. Some hydroid larvae settled on small, slender tubes representing juvenile forms of serpulids, which indicate that bioclaustration took place in the early ontogenetic stages of the worm. Another putative explanation is a random acquisition of the host, which might have resulted in the recruitment of only those larvae where “the good” choices have been made, whereas most of them, in all likelihood, never develop. The substantiation of such a mechanism is that the capability of free-living larvae to control their movement is very limited (see [88]). Recent bioclaustrated symbionts also show host preferences ([20, 89, 90]; however, see [91]), and some of them are completely dependent on the host [92].

Possible interpretations of hydroid-serpulid symbiosis involve mutualism, commensalism, and parasitism. Due to the large host’s selectivity of *Protulophila gestroi* as well as a lack of evidence for serpulid deriving benefits from this interaction (except the supposed protection provided by the hydroids’ nematocysts), commensalism appears to be the most plausible kind of relationship. Furthermore, this type of interaction demonstrates a strong host preference leading to some obligate host-colonizer cohabitants [20], which might at least partially explain the selectivity of the hydroids.

Both mutualism and parasitism between *Protulophila gestroi* and *Propomatoceros* seem to be unlikely. In the first case, as already mentioned above, we don’t have any evidence that *Protulophila* possessed nematocysts which could have protected the serpulid host. Additionally, the percentage of infestation is too low to consider mutualism as a confident type of interaction–hydroids probably have not been notably advantageous for serpulids. In the second case, although the assumption that the incorporation of hydroids bears energy expenditure for serpulids may be reasonable, nothing indicates that the bioclaustrated hydroids might have been parasites. In both cases, there are no disparities between the infested and non-infested serpulids of the genus *Propomatoceros* (and other genera), resulting either in the exhibition of particular patterns by serpulids, which might have provided any successful ecological solutions (mutualism), or in malformations of the polychaetes’ tubes and conceivably smaller size and/or slower skeletal growth of the host due to the harmful activity of the hydroids. Moreover, symbiotic relations can range (shifting during ontogeny) from mutualistic to parasitic and *vice versa* (e.g., [93]), which makes this assessment even more difficult. Even if the explanation of hydroid cnidocytes’ protective contribution (whichever way possible) is true, in all likelihood, it did not play any significant role. A fully confident determination of the relationship between mutualistic, commensal, and parasitic is not easy in the fossil record. In the present case, however, a commensal relationship, even though it is still difficult to prove in the fossil record (see [94]), is the most plausible and acceptable on the grounds of the available evidence presented above.

### How common was the serpulid-hydroid symbiosis in the marine Jurassic Polish Basin?

Generally, the presence of *Protulophila gestroi* in the fossil record coincided with the growing diversification of serpulids that started during the Late Triassic-Lower Jurassic and continued during the entire Mesozoic [95]. Thus, this symbiotic relationship may have originated somewhere during the Triassic–Jurassic transition, as the oldest example comes from the Pliensbachian [25]. An increase in the abundance of *Protulophila gestroi* over time appears to be related to two factors: 1) higher taxonomic diversity and abundance of serpulid fauna in the Cretaceous [96], whose skeletons provided a suitable substrate for colonization and 2) generally better taxonomic recognition of Upper Cretaceous serpulid worms compared to the Jurassic ones, a factor which increases a chance for detection of hydroid symbionts in a larger collection of host serpulids.

The percentage of infestation cases in the present study (0.6% among all 3,346 well-preserved fossils, see Table 1) was much lower than in most settings previously investigated, where the rates may even reach 45% [15] or 50% [86]. However, despite several reports of *Protulophila gestroi*, its paleoecology and the rate of infestation have rarely been studied, as the main scope of the research was polychaete fossils and not hydroids (e.g., [14, 25, 27]), or the study was based on a single specimen only [29]. The infestation rate seems to be commonly higher in younger, Cretaceous deposits [15, 18, 86]. Except for very few reports of this association from the Jurassic [8, 14, 26], no complex research has been conducted to better understand the abundance and paleoecology of this relationship. Reports on this association from the Cenozoic are also rarer than those from the Cretaceous. Despite the growing diversity in Palaeogene, serpulid fauna has been relatively poorly studied from this stratigraphic interval (see [95]), which also possibly reflects very few reports on the serpulid-hydroid relationship from this time interval [8, 22, 85]. The lack of comprehensive reviews of the hydroid-serpulid coexistence in the Jurassic may result from a significantly lower percentage of infestation, which may, in turn, be a consequence of unfavorable conditions for *Protulophila gestroi* to settle.

It has to be highlighted that most of the collected specimens of bioclaustrated hydroids (17 cases per 187 well-preserved serpulids) were derived from a single stratigraphic interval (Gnaszyn Dolny, Middle Bathonian, Morrisi Zone, Table 2) representing deeper (transgressive cycle T5 of [50]), calm paleoenvironment characterized by a muddy bottom on which a hard substrate suitable for serpulid colonization was very patchy or completely absent. There, 17 hydroid infestation cases have been noted in *Propomatoceros lumbricalis*, which constitute 9% of the all 187 well-preserved serpulids collected (Table 2). However, the infestation percentage could even be higher here, if the rest of 74 *Propomatoceros* tubes weren’t worn. The single cases of infestation of *Propomatoceros* tubes noted in the remaining assemblages (Table 2), certainly result from a much lower number of perspective specimens. In Gnaszyn Dolny, hydroids have been found to infest serpulid tubes located on the same, very small substrate surfaces provided by oyster shells (Fig 6). This may favor the explanation that due to the very limited mobility of hydroid larvae, most of them presumably have not been able to recruit, unless settled on a convenient substrate (serpulid tube) providing bioclaustration (protection of colonies) and possibly also nutrient supply. In the case of the successful settlement of hydroid larvae on a given serpulid, their further spread was strongly limited by the available hard substrate with other potential hosts. Thus, the hydroid larvae had the greatest chance to colonize the neighboring serpulids on the same substrate, as exemplified by those present on the single oyster shells (Fig 6). The colonization of other hosts, growing on separate substrates, might have been exceedingly difficult or impossible for the larvae. The lack of any hydroid symbionts noted in abundant serpulids, including *Propomatoceros*, colonizing the Middle and Upper Bathonian hiatus concretions from Bugaj, Ogrodzieniec, and Krzyworzeka [26], or large oncoids from Ogrodzieniec [56] is striking. However, in these cases, the paleoenvironmental conditions might have, at least to some extent, played a role. The hiatus concretions and oncoids were repeatedly overturned on the seabed due to hydrodynamical processes and/or animal activities (e.g., [26, 48, 56]); thus, they might not have provided a sufficiently stable habitat for the host serpulids and thus for the colonizing hydroids, as well. Even though some larvae may have been recruited on some serpulid hosts, they could not have developed when substrate with the hosts were overturned. Additionally, stronger currents in such settings might have prevented the hydroid larvae from settling on the hosts.

The lack of any bioclaustrated hydroids within the polychaete tubes from the Oxfordian sponge buildups of Zalas may be caused by a lack of suitable specific species of the host. In the present study, the polychaete tubes are represented by dominating *Glomerula*, followed by *Propomatoceros* and *Tetraserpula* (*Nogrobs*), constituting 75%, 24.8%, and 0.2% of the polychaete assemblage, respectively (as calculated from Kuziomko-Szewczuk [71]). There, either 1) the hydroids have not been present or 2) did not develop following settlement on the host, or 3) these polychaete species were not suitable hosts for the symbionts. Interestingly, in the Oxfordian of central Poland, Radwańska [14] noted only one example of *Protulophila gestroi* preserved within the tube of *Ditrupula*, a genus not found in the Oxfordian assemblage of Zalas. A similar situation might have been responsible for the lack of any *Protulophila gestroi* specimen preserved within the polychaete tubes from the Kimmeridgian of Małogoszcz, which are represented by similar forms as in the Oxfordian of Zalas (see [70]). In summary, the fossil record of serpulid-hydroid symbiosis within the Polish Basin during the Middle and Late Jurassic was a rare phenomenon with a very patchy distribution, mostly limited to a single setting characterized by a calm paleoenvironment with a slow (or even halted) sedimentation rate, allowing for the establishment and persistence of a suitable hard substrate for hydroid hosts.

## Conclusions

The overall appearance of a hydroid colony is shown by external, subcircular apertures with bosses of different shapes and sizes and an internal network of branching stolons and polyp chambers. The morphology of the bioclaustrated traces left by hydroids shows a very independent arrangement of the colony, both within a given stratigraphic interval and through time. The differences in morphological details occur regardless of the geological interval, as it presumably reflects physiological and paleoecological conditions rather than interspecific variability. Although there is a faint probability that all the hydroid remnants described hitherto spanning through ca. 190 Ma belong to the single species *Protulophila gestroi*, we are not able to provide any reliable lower taxonomic classification based exclusively on the bioclaustrated traces as morphological disparities occur within all stratigraphic intervals. In order to make better comparisons of different colonies for any future taxonomic classification, a micro-CT scanning and volumetric rendering providing a number of features not visible externally, would be a good solution.

The studied hydroids show a significant bias toward certain serpulid genera. It possibly reflects some favorable conditions for *Protulophila gestroi* recruitment, comprising chemical composition, sufficient protection, and nutrient supply. The dependence on certain paleoecological conditions is striking, as current findings are almost restricted to only one stratigraphic zone, representing a specific paleoenvironment. The mobility of larvae might have been poor, which is also reflected in accumulated hydroid occurrences on a very small substrate. If convenient recruitment conditions existed, hydroids colonized close neighboring tubes and might not be able to colonize more distant, separated substrates. Based on all the available data, the most probable type of hydroid-serpulid symbiosis is commensalism.

The frequency of bioclaustrated hydroids is scarce in the material studied with a percentage of infestation of 0.6% among the well-preserved polychaete fossils. Such a pattern of occurrence of serpulid-hydroid symbiosis during the Middle and Late Jurassic within the Polish Basin seems to have resulted from an interplay of biological (host specificity, mobility of symbiont larvae and their survival) and paleoenvironmental (hydrodynamism and sedimentation rate) factors. The presence of hydroid symbionts only within the tubes of a single genus *Propomatoceros* indicates that host specificity plays an important role in the hydroid larvae. Taphonomy (preservation of tubes) probably played a minor role, as well-preserved tubes in a given assemblage analyzed here constituted a fairly large sample size.

The present case study shows that serpulid polychaete-hydroid symbiosis over a long-time interval within a single basin may show a very patchy distribution, concentrated only in single intervals where appropriate conditions for its development occurred. The morphological stasis of the preserved structures after hydroid *Protulophila gestroi* combined with its rarity in many stratigraphic intervals precludes any firm analyses concerning the co-evolution of both symbionts in both time and space of the same paleogeographic entity.

## Supporting information

S1 Movie CT longitudinal sectioning of specimen GIUS 8-3730/3 showing internal appearance of serpulid-hydroid association where polyp chambers and connected with them branching stolonal tubes are visible. (AVI)

S2 Movie Visualization of the individual tube of the serpulid *Propomatoceros lumbricalis* infested by hydroids *Protulophila gestroi*. (AVI)
